# Editorial: Liver cancer awareness month 2023: current progress and future prospects on advances in primary liver cancer investigation and treatment

**DOI:** 10.3389/fonc.2024.1453709

**Published:** 2024-07-01

**Authors:** Francisco Tustumi, Rodrigo Xavier das Neves, Marina Alessandra Pereira, Fabricio Ferreira Coelho, Wellington Andraus

**Affiliations:** ^1^ Department of Gastroenterology, Universidade de São Paulo, Sao Paulo, SP, Brazil; ^2^ Department of Surgery, Hospital Israelita Albert Einstein, Sao Paulo, SP, Brazil; ^3^ National Cancer Institute, Center for Cancer Research, Bethesda, MD, United States; ^4^ Department of Gastroenterology, Instituto do Cancer do Estado de São Paulo, Sao Paulo, SP, Brazil

**Keywords:** liver neoplasms, hepatocellular carcinoma, cholangiocarcinoma, hepatectomy, liver cancer

## Introduction

October is Liver Cancer Awareness Month, and at Frontiers in Oncology, we highlighted recent discoveries in the field and raised awareness about the importance of early diagnosis, multidisciplinary management, and technological innovation in supporting liver cancer treatment. Primary liver cancer presents a significant global health challenge. Liver cancer poses substantial morbidity and mortality rates. Wang et al. reported a crude incidence of liver cancer of around 26/100,000 and a mortality of 22/100,000 for the year 2020. The leading primary liver cancers comprise hepatocellular carcinoma (HCC) and cholangiocarcinoma, while other neoplasms, such as primary hepatic adenosquamous carcinoma, are rare [Ai et al.] (1).

**Figure 1:**
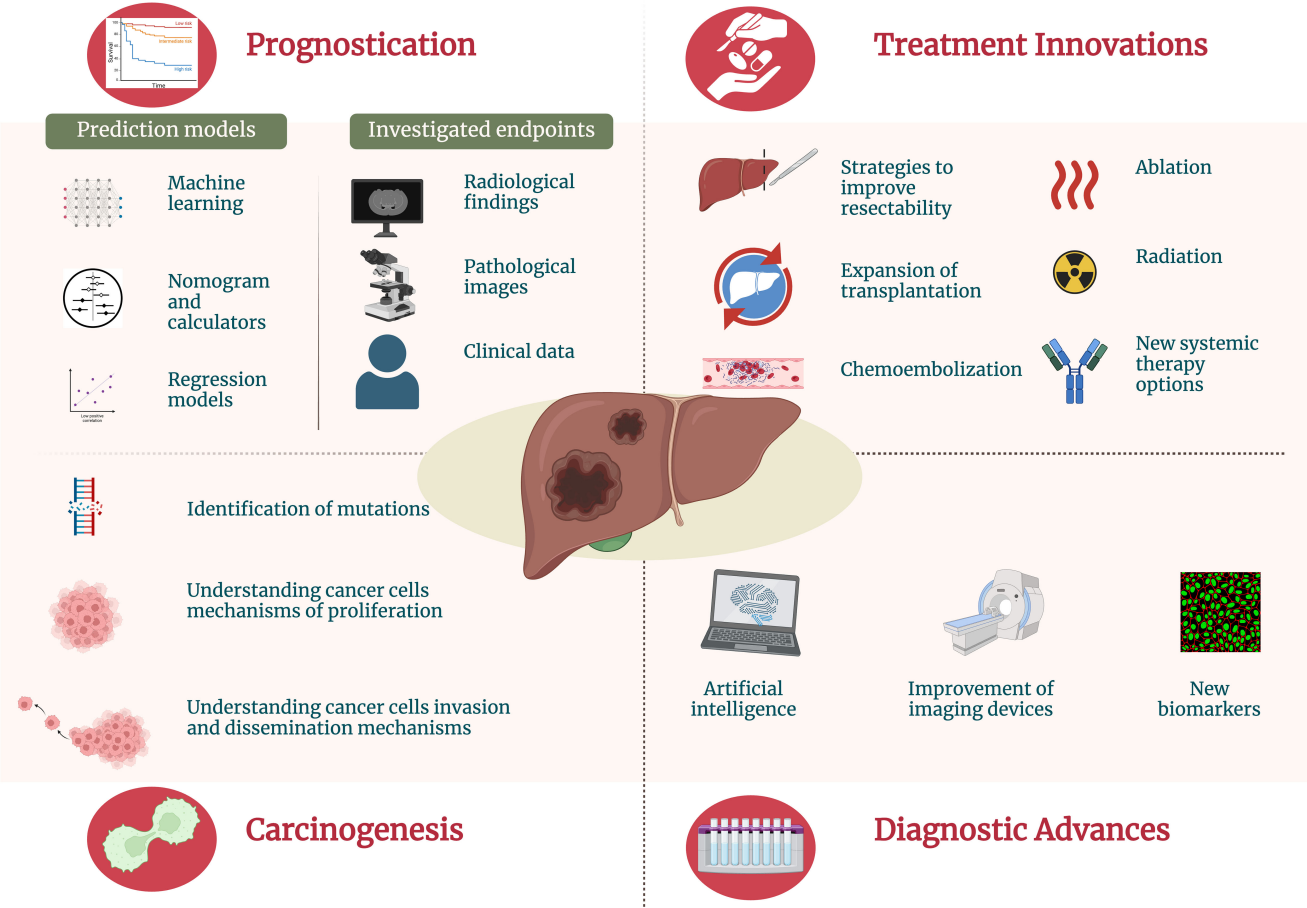
Current advances and innovations for primary liver cancer management.

This Research Topic focuses on the latest advancements in investigating and treating primary liver cancer, providing insights into cutting-edge approaches that shape the field and improve patient outcomes (see **Figure 1**).

## Carcinogenesis

Chronic liver disease and viral hepatitis, especially hepatitis B, work as preneoplastic conditions due to the increased risk for primary liver cancer transformation. Guo et al. identified the variables age, sex, antiviral therapy history, hepatitis B antigen, alcohol drinking history, and serum alpha-fetoprotein levels as risk factors for cancer HCC development in patients with cirrhosis. Recent advances in the carcinogenesis of primary liver cancer help us understand how risk factors can lead to cancer development and progression (2). By knowing these pathways, it is possible to interfere in crucial steps of carcinogenesis, reducing the risk for cancer development and working as a target for new cancer therapies. Continuous liver damage and regeneration lead to cellular mutations and malignant transformation. Recent molecular investigations have identified several key pathways involved in cancer development for hepatocellular carcinoma and cholangiocarcinoma, including the Wnt/β-catenin, PI3K/AKT, and MAPK signaling pathways, which play critical roles in cell proliferation, survival, and apoptosis (3, 4). Other factors, such as excessive alcohol consumption and exposure to toxins, also play critical roles in liver carcinogenesis by direct DNA damage. Recent molecular studies have highlighted the importance of epigenetic changes, such as DNA methylation and histone modification, in the development and progression of HCC (5).

Understanding these risk factors and the associated molecular pathways is crucial for developing targeted prevention and early detection strategies in high-risk populations, as well as for identifying potential therapeutic targets for treating HCC.

## Prognostication

Prognostication in oncology is a dynamic area of research that aims to refine our understanding of disease progression and patient outcomes. Prognostication refers to the process of predicting the likely course and outcome of the disease (6). It involves using predictive models to assess prognostic variables, such as the type and stage of cancer, tumor characteristics, patient health status, and endpoints, such as response to treatment and survival rates. This information is crucial for guiding treatment decisions, setting realistic expectations, and planning follow-up care. Accurate prognostication helps healthcare providers tailor interventions to individual patients, ultimately aiming to improve outcomes and quality of life. In fact, the main scores for liver function (which heavily influences liver cancer treatment), such as Child-Pugh and Model for End-Stage Liver Disease, are based on prognostic indicators of survival (7, 8). Prognostic calculators or nomograms are helpful because they can be easily used in clinical practice. Tian et al. performed a retrospective analysis of HCC patients. The authors used regression models to construct a predictive nomogram based on the following independent prognostic indicators of disease-free survival: major resection, albumin, microvascular invasion, laparoscopic surgery, blood loss, bilirubin, and pleural effusion. A study by Sun et al. estimated the tumor burden based on the sum of tumor numbers and maximum diameters. Their results highlight how tumor burden influences progression patterns and survival outcomes in patients under sorafenib treatment, emphasizing the need for tailored treatment strategies. Tan et al. built a novel immune classification based on the immune infiltration within the tumor microenvironment using pathological images to predict early HCC recurrence, offering a valuable tool for identifying high-risk patients.

However, scores based only on regression models can be limited since they only comply with a handful number of clinical or laboratory variables. This Research Topic is especially relevant since liver conditions comprise multifactorial prognostic variables. In this sense, lately, the use of artificial intelligence has boosted the predictive capability for estimating liver cancer prognostication. A machine learning approach for personalized prognostic assessment further enhances our ability to predict patient outcomes and tailor treatments accordingly. Zhang et al. created a multi-level prognostic risk model for HCC. Their models exhibited a high performance in predicting patient response to therapy.

## Diagnostic advances

Early and accurate diagnosis of primary liver cancer is crucial for effective treatment. Innovations in diagnostic methods are improving our ability to detect primary liver cancer at earlier stages. The diagnosis of liver neoplasms frequently relies heavily on imaging tests. Using serum markers, such as alpha-fetoprotein (AFP), is helpful to increase the accuracy of liver cancer diagnosis. However, some tumors do not express AFP, puzzling liver cancer diagnosis. In this sense, serum interleukin-41 has emerged as a novel serum marker for diagnosing AFP-negative HCC [Li et al.]. In addition, interleukin-41 can also serve as a prognostic marker for HCC.

The construction of diagnostic models using machine learning has also shown promise in enhancing the accuracy of HCC progression detection. Jiang et al. utilized machine learning techniques to construct diagnostic models for HCC across different stages of the disease progression. Fu et al. also used machine learning to improve the diagnostic accuracy for preoperative differentiation between xanthogranulomatous cholecystitis and gallbladder carcinoma.

Advanced imaging techniques are revolutionizing not only liver cancer diagnosis but also staging. Pretreatment determination of vascular invasion is crucial in primary liver cancer since it impacts a patient’s prognosis and highly influences treatment. Pan et al. evaluated perfusion indexes and spectral parameters to diagnose portal vein tumor thrombus. Yu et al., using a deep learning approach, created models to enhance preoperative diagnosis of microvascular invasion through domain-adaptation fusion of multi-phase CT images.

## Treatment innovations

Treatment options for primary liver cancer are rapidly evolving, with a focus on personalized and multimodal approaches. While the transplantation is well-established for HCC, the use of transplantation for other primary liver cancers is not well-studied. However, recent studies have shown promising results for transplantation in cholangiocarcinoma, expanding the current indications for liver transplant [Andraus et al.] (9).

With the latest advances in liver surgery and postoperative care, liver resection has also expanded, and tumors once considered unresectable, nowadays are being treated with curative intention. Martinino et al., in a systematic review, found that liver resection for HCC presents similar long-term survival than transplantation if an appropriate patient selection is performed.

Currently, there is still debate about the best approach for HCC with tumor thrombus, but it seems that surgical alternatives (liver resection or transplantation), if feasible, have better outcomes (10). However, other treatment strategies should be considered for patients who are not candidates for surgery. Leung et al. evaluated multimodal strategies for advanced hepatocellular carcinoma with portal vein tumor thrombus. The authors found that the hepatic arterial infusion chemotherapy of fluorouracil, leucovorin, and oxaliplatin, with or without sorafenib, demonstrated superior survival rates than alternative treatments.

Thermal tumor ablation is a minimally invasive locoregional therapy that eradicates tumors by applying heat to eliminate malignant cells. This technique comprises radiofrequency ablation and microwave ablation (11). These techniques might be challenging to apply in certain difficult locations within the liver, such as proximity to other organs, such as the gastrointestinal tract, diaphragm, or gallbladder, due to the risk of internal bleeding or iatrogenic injury (12). In these cases, the application of hydrodissection in microwave ablation can be helpful, by separating the tumor from nearby health tissues, with a success of over 90% [Song et al.].

A systematic review and meta-analysis [Dong et al.] compared repeat hepatectomy and thermal ablation therapy for recurrent HCC. The authors found that this approach was related to fewer complications due to the less invasiveness of thermal ablation. However, the overall survival and the recurrence-free survival were higher for those treated with repeated hepatectomy. Consequently, the reduced complication rate of thermal ablation allows for quicker patient recovery and shorter hospital stays, making it a potentially safer option for individuals who cannot tolerate major surgery.

For those HCC patients who are not candidates for surgery, treatment strategies are usually based on systemic therapies. Tyrosine kinase inhibitors such as sorafenib or lenvatinib are the most common drugs used in these patients. However, currently, multimodal therapeutic strategies are being studied (13). Combining traditional treatments with newer agents showcases the potential for synergistic therapeutic effects. Chen et al., in a retrospective controlled study, compared transarterial chemoembolization in monotherapy *versus* transarterial chemoembolization associated with anlotinib, a novel oral multi-kinase inhibitor. The group treated with anlotinib showed higher survival rates.

In the last decade, target therapy and precision medicine have revolutionized HCC management. Han et al. in a multicenter, propensity-score-matched study, compared atezolizumab-bevacizumab *versus* lenvatinib in HCC. The authors found that the combination atezolizumab-bevacizumab is a promising treatment strategy for advanced HCC, with better overall survival than lenvatinib. Jiang et al. described their treatment strategy for advanced HCC using hepatic arterial infusion chemotherapy with immunotherapy.

## Conclusion

This Research Topic provided a comprehensive overview of recent advancements in investigating and treating primary liver cancer. Recent advances in the knowledge of primary liver cancer carcinogenesis, prognostication, diagnosis, and treatment are essential aspects for improving liver cancer patient care.

## Author contributions

FT: Conceptualization, Writing – original draft. RX: Investigation, Validation, Visualization, Writing – review & editing. MP: Data curation, Visualization, Writing – review & editing. FC: Supervision, Writing – review & editing. WA: Supervision, Writing – review & editing.
